# Reply to Xiao and Galstyan: Reliable ligand discrimination involves many trade-offs

**DOI:** 10.1073/pnas.2411465121

**Published:** 2024-07-25

**Authors:** Duncan Kirby

**Affiliations:** ^a^Department of Physics, University of Toronto, Toronto, ON, M5S 1A7, Canada

The letter by Xiao and Galstyan briefly analyzes how varying the time a cell has to discriminate between ligands impacts reliable ligand discrimination in the canonical kinetic proofreading problem with stochasticity taken into account (1). The authors expand on the work of Kirby and Zilman, which investigated how both the proofreading strength and the number of proofreading steps affect stochastic noise in molecular counts and the resultant reliability of ligand discrimination (2). Xiao and Galstyan relax the assumption of a fixed discrimination time in the previous work to show that the issue of stochastic noise in molecular counts can be alleviated by sufficiently increasing the discrimination time. It is already clear from inspection of equations 1 and 2 in ref. 2 that, in the case of a fixed number of proofreading steps, increased discrimination time can improve resolution in this discrimination task because the distributions of signaling molecule counts for different ligands separate monotonically with time. Other aspects of the trade-off between proofreading strength, proofreading steps, and the variation in the distribution of signaling times for stochastic kinetic proofreading have also been investigated elsewhere (3, 4).

Xiao and Galstyan provide analysis in figure 1 which shows that discrimination performance can improve monotonically with *N*, the number of proofreading steps, when the discrimination time also increases as a power of *N*. However, the requirement for a geometric scaling of the discrimination time could be a significant limitation in many cellular decision processes. For example, antigen detection by T cells is limited to a few minutes because this is the time a T cell spends scanning the surface of an antigen-presenting cell (5). A large increase in the discrimination time may not be compatible with such a limitation. In other scenarios, cellular decision time may be quite flexible and the fixed activity setup which the authors consider may be quite reasonable. Interestingly, the timescale of some cellular decision-making processes—including T cell antigen detection—may change dynamically with other environmental factors (5, 6). This only reinforces the conclusions of Xiao and Galstyan that further study of the interplay between discrimination time, discrimination accuracy, and signaling activity would be beneficial for the field.
